# Gestational syphilis and prenatal care: a study in light of individual, social, and programmatic vulnerabilities

**DOI:** 10.1590/1980-220X-REEUSP-2025-0270en

**Published:** 2026-05-29

**Authors:** Adriana de Oliveira Sarefino, Ana Beatriz Azevedo Queiroz, Karla Gualberto Silva, Norma Valéria Dantas de Oliveira Souza, Natalia Moreira Leitão Titara, Ana Luiza de Oliveira Carvalho, Juliana da Fonsêca Bezerra, Ana Kedma Correa Pinheiro, Aline Furtado da Rosa

**Affiliations:** 1 Universidade Federal do Rio de Janeiro, Escola de Enfermagem Anna Nery, Rio de Janeiro, RJ, Brazil.

**Keywords:** Syphilis, Sexually Transmitted Diseases, Health Vulnerability, Primary Health Care

## Abstract

**Objective:**

To analyze the perception of women diagnosed with gestational syphilis regarding prenatal care within the Family Health Strategy and to discuss the individual, social, and programmatic vulnerabilities related to this context.

**Method:**

Qualitative and descriptive research, conducted with 20 women diagnosed with gestational syphilis, reported at two Family Clinics located in the Santa Cruz neighborhood, Rio de Janeiro/Brazil. Data collection was carried out through individual semi-structured interviews in the participants’ homes, and data processing was performed using the software IRAMUTEQ.

**Results:**

The participants were mostly mixed-race or black, with low levels of education, unemployed, and with low income, indicating social vulnerability. Syphilis was primarily diagnosed during pregnancy, with some cases only identified during childbirth or postpartum. The results pointed to individual, social, and programmatic vulnerabilities related to lack of knowledge, barriers to access, and failures in prenatal care.

**Conclusion:**

Gestational syphilis is associated with social and structural factors that require integrated actions in health education, strengthening prenatal care, and expanding the involvement of partnerships in treatment.

## INTRODUCTION

Gestational syphilis (GS) represents a significant challenge for global public health, especially in underdeveloped or developing countries, as is the case in several nations of the Americas. Early identification of GS and the implementation of clinical protocols during prenatal care, childbirth, and the postpartum period are essential to reduce the incidence of congenital GS infections^(1)^. Congenital syphilis (CS) can cause malformations and bone abnormalities, among other early and late complications and manifestations, such as miscarriage, premature birth, neonatal death, low birth weight, neurological impairment, including intellectual disability and hearing impairment^(2)^.

In the Americas, the percentage of pregnant women with syphilis has increased by 28% in the last two years, resulting in a proportional increase in cases of CS, reaching an estimated 4.98 cases per 1,000 live births in 2022, a figure that significantly exceeds the target set by the World Health Organization (WHO) of 0.5 cases per 1,000 live births^(1)^. According to the WHO^(3)^, every year, GS accounts for more than 300,000 fetal and neonatal deaths worldwide, in addition to putting more than 200,000 children at risk of premature death. In Brazil, cases of acquired syphilis, GS, and CS have shown a significant increase in recent years, highlighting the need for effective and comprehensive interventions in prenatal care, taking into account situations of vulnerability^(2)^.

Prenatal care represents the best opportunity for GS screening, diagnosis, treatment, and prevention, both for pregnant women and their partners. Early diagnosis and timely treatment of syphilis in pregnant women are crucial steps in reducing vertical transmission and preventing complications. It is recommended that pregnant women be tested at least at their first prenatal visit, preferably in the first trimester of pregnancy, at the beginning of the third trimester, and at the time of admission for delivery. When diagnosed, they should be treated immediately, and continuity of care should be ensured by the healthcare team^(2)^.

In Brazil, the Family Health Strategy (FHS), within the context of Primary Health Care (PHC), plays a fundamental role in expanding access to and promoting quality prenatal care, being considered an essential strategy for the control and prevention of GS and CS^(4)^.

Although access to prenatal care is largely guaranteed in Brazil, a study points to gaps in the quality of care provided, which generates worrying situations of vulnerability^(5)^. Research conducted in Rio Grande do Sul showed that, even among pregnant women who received prenatal care, inadequate (52%) and absent (32.4%) treatments remain among the vulnerability factors for CS, as does the lack of treatment of sexual partners, which is one of the main obstacles to the effective control of syphilis^(6)^.

The lack of access to quality healthcare services, especially regarding screening and effective therapeutic interventions, remains a significant vulnerability factor for the high prevalence of this condition in various regions of the world^(7)^.

Although GS is not among the 20 neglected tropical diseases, it is included in the strategies and goals established by the WHO^(8)^ to eliminate negligence and achieve the Sustainable Development Goals (SDGs) by 2030. International scientific literature, however, recognizes GS as a neglected disease^(9)^, strongly associated with conditions of vulnerability affecting specific populations^(10,11)^.

It should be emphasized that the definition of vulnerability relates to a set of individual, collective, and contextual aspects that lead to greater susceptibility to infection and illness, and is inseparable, to varying degrees, from access to resources to ensure protection^(12)^.

Health vulnerability is understood as a human condition resulting from the interaction between the individual and society, based on power relations, and which can be precarious when the individual’s or the community’s empowerment is not being experienced at the moment^(13)^. These vulnerabilities involve sociodemographic aspects, including access to health services, socioeconomic conditions, and the cultural dimension, which originate from society itself and interfere with the movement of individuals and groups within a social hierarchy^(11)^.

National and international studies show that unfavorable social determinants, such as low education levels, reduced income, and migration, as well as a deficit in health education, contribute to greater vulnerability to GS and its consequences^(14,15)^. In this context, vulnerability analysis becomes fundamental to understanding the factors that influence the **high incidence and prevalence rates**, the persistence of GS, and the occurrence of vertical transmission. These vulnerabilities can be understood from three interdependent dimensions: the individual one, which involves biological, behavioral, and emotional aspects; the social one, related to context and interpersonal relationships; and the programmatic one, which concerns public policies, programs, and available health services^(16)^.

These dimensions encompass individual aspects related to exposure to and susceptibility to the harm in question; contextual characteristics and established social bonds; as well as how technologies expressed in policies, programs, services, and actions operate in these contexts and influence the situations^(16)^. Thus, these three analytical dimensions contribute to understanding the relationship between the individual and vulnerability.

To effectively address GS, it is critical to identify the factors related to vulnerabilities that favor the persistence of GS and to implement public health strategies that consider these vulnerabilities and promote equitable access to health services. Therefore, an analytical framework is required that incorporates individual behavior, the collective sphere, and the social context^(16)^.

Considering this issue, the following question arises: what is the perception of women diagnosed with GS regarding the prenatal care provided in the FHS and the possible individual, social, and programmatic vulnerabilities associated with this care?

The relevance of this study is justified by the impact of CS in the Brazilian setting, considering the immediate and long-term consequences for newborns and neonatal health. In this context, the present study aims to analyze the perception of women diagnosed with GS regarding prenatal care in the FHS and to discuss individual, social, and programmatic vulnerabilities.

## METHOD

### Study Design

This is a descriptive study with a qualitative approach, guided by the guidelines of Consolidated Criteria for Reporting Qualitative Research (COREQ)^(17)^.

### Population, Local and Selection Criteria

The research was conducted in Planning Area (*AP*) 5.3, located in the western zone of the municipality of Rio de Janeiro/Brazil, in the homes of women reported with GS at two Family Clinics located in the Santa Cruz neighborhood. It is worth highlighting that the selection of this area for the research is justified by the fact that it is an area with a high incidence of CS cases, according to data extracted from the Notifiable Diseases Information System (*SINAN*). The research was conducted exclusively by the researcher, a nurse working in PHC and familiar with the territory studied, which facilitated closer relationships with the participants and a better understanding of their reported experiences. Contact with the participants was conducted in an ethical, empathetic, and respectful manner, ensuring the confidentiality of information and analytical neutrality in the interpretation of results.

Twenty women who had been diagnosed with GS participated in the study, meeting the inclusion criteria: minimum age of 18 years, who had prenatal care at the FHS of *AP* 5.3, and who had GS notifications between the years 2016 and 2021. This period corresponds to the implementation of the Strategic Action Agenda for Syphilis Reduction in Brazil, which prioritized the integration of teaching and research institutions to expand strategies for tackling the disease^(18)^. Women reported in *SINAN* who did not reside in the study area were excluded.

### Data Collection

Data collection took place between August 2023 and March 2024, and the selection of participants was based on the table of reported cases provided by the Health Surveillance Division of the PHC Coordination of *AP* 5.3. The table in question contained a total of 67 women and some information such as age, address, and telephone number. Of that total, 19 did not meet the inclusion criteria, 13 no longer lived at the address provided, four did not want to participate in the survey for personal reasons, and 11 did not answer phone calls. Therefore, home visits were scheduled with 20 participants, according to the women’s preferred days and times, and data collection was carried out in the participants’ homes, in individual rooms to ensure information privacy, with only the researcher present.

The data collection technique used was a form with closed-ended questions to outline the sociodemographic characteristics and data related to GS diagnosis. The instrument contained the following data: age, education level, self-declared race/color, religion, profession/occupation, family income, and social benefits. Regarding the diagnosis of GS, the data collected were: gestational age at the start of prenatal care, number of consultations, period of syphilis diagnosis, treatment performed, and treatment of sexual partners.

The second stage of data collection was an in-depth individual semi-structured interview covering the following topics: perception of prenatal care after a syphilis diagnosis during pregnancy, what it was like to receive the diagnosis, what explanations/guidance regarding GS were like, and perception of access to health services and follow-up in relation to diagnosis, GS treatment, and CS prevention. It should be noted that two pilot interviews were conducted with pregnant women diagnosed with CS who did not meet the inclusion criteria for the time frame, in order to make adjustments to the interview script, but these were not included in the total sample size.

The interviews were recorded, after the reading and signing of the Free Informed Consent Form, with authorization, using an application on a mobile device, and had an average duration of 30 minutes and, subsequently, were transcribed in full for analysis.

### Data Analysis and Treatment

The data analysis process for the interviews was carried out using the software IRAMUTEQ (*Interface de R pour les Analyses Multidimensionnelles de Textes et de Questionnaires*), through lexical content analysis. This software allows the integration of statistical methods and subjective analyses, with easily interpreted graphical representations applicable to large databases, provided that the textual corpus is properly prepared^(19)^. The textual corpus was organized, encompassing the concepts of corpus, text, and text segment (TS). The corpus encompassed all 20 interviews, each treated as a text, with the TS defined as an analytical unit of approximately three lines, automatically adjusted by IRAMUTEQ^(19)^.

The command lines, preceded by four asterisks (****) and followed by specific codes, identified the participants using the letter M for woman (*mulher* in Portuguese) followed by the numeral (M_1 to M_20). The variables studied were age, having a partner, undergoing treatment, and education. All these steps ensured methodological rigor, the anonymity of the participants, and the organization necessary for data processing and analysis.

This study used Descending Hierarchical Classification (DHC) analysis, which groups TSs based on similar vocabularies, fragmenting the textual corpus according to the frequency of reduced forms^(19)^. The main objective is to identify vocabulary classes presenting internal homogeneity and heterogeneity among themselves, allowing for detailed TS analysis through Chi-square (chi^2^) tests to calculate distances and proximities between terms^(19)^.

The DHC analysis divided the textual corpus into five thematic classes, totaling 81.01% utilization of the analyzed material. This breakdown revealed two thematic blocks, with block 1 entitled “Gestational syphilis and prenatal care: the presence of vulnerabilities,” consisting of classes 1, 4, and 5. This section addressed the objective of this study, focusing on aspects related to the identification of syphilis during pregnancy, the participants’ perception of the care received, and individual, programmatic, and social vulnerabilities in prenatal care.

The raw results provided by the software were processed and interpreted, seeking to assign meaning and validity to the classes found. The analysis included logical deductions, description, and contextualization based on relevant scientific literature, allowing for a more in-depth interpretation of the discourses and an understanding of the implicit messages in the identified vocabularies, which were analyzed and named based on theoretical frameworks on GS and dimensions of vulnerabilities.

### Ethical Aspects

The study was conducted in accordance with Resolution No. 466/2012 of the National Health Council/Ministry of Health, which regulates research involving human beings, and with Resolution No. 580/2018, applicable to research carried out in institutions of the Brazilian Public Health System (*SUS*). It was reviewed and approved by the Research Ethics Committee of the Universidade Federal do Rio de Janeiro – Anna Nery School of Nursing/São Francisco de Assis Teaching Hospital, CAAE 66485023.2.0000.5238 and opinion number 6.175.541, and by the Research Ethics Committee of the Municipal Health Department of Rio de Janeiro, CAAE 66485023.2.3001.5279 and opinion number 6.210.457.

## RESULTS

The participants in this study were 20 women, aged between 20 and 48 years, with the 30-39 age group being the most prevalent (nine women). Regarding self-declaration of race/color, 13 identified themselves as mixed-race and five as black, totaling 18 black women. Educational levels were predominantly low, with eight women having unfinished secondary education. Regarding religion, 12 stated they had no religious bond, while eight stated they practiced Christian religions, six being evangelical and two Catholic. Regarding marital status, nine were single without partners, while 11 lived with partners. Among the single women, seven lived only with their children, while the women with partners mostly resided with their husbands and children. The occupation analysis revealed that 14 were unemployed and six were engaged in paid activities. Family income ranged from less than one to four minimum wages, with 10 surviving on less than one minimum wage. Furthermore, 13 were receiving some form of social benefit from the government.

In prenatal care, 12 patients began prenatal care before 12 weeks of gestation, and six of them did not remember the gestational age at the start of their care. The number of consultations ranged from four to 14, with nine women having fewer than six consultations, a number lower than recommended by the Ministry of Health^(2)^. Additionally, syphilis was diagnosed mostly during pregnancy in 15 women, while five were diagnosed only at the time of delivery or after their child’s birth. Among the pregnant women diagnosed, 15 underwent treatment with Benzylpenicillin, two used other medications due to allergies, and one received no treatment. Regarding sexual partners, 12 stated that their partners underwent treatment, while 8 participants stated that their partners did not agree to use the medication.

In the results of the interviews, regarding the analysis of the DHC of the software IRAMUTEQ, for this article, the classes corresponding to block 1, classes 1, 4 and 5, were analyzed and organized according to the vulnerabilities presented.

Thus, the classes generated by IRAMUTEQ were interpreted in light of the three dimensions of vulnerability in health: individual, social, and programmatic. Thus, a single class can contain content that engages with more than one dimension, being presented according to the analytical focus that predominates in each axis.

### Individual Vulnerability

#### Class 1 – Diagnosis of Gestational Syphilis and (Mis) Information: The Unexpected Revelation

Class 1 consisted of 9 TSs, representing 14.06% of the material classified for analysis. The lexical variables diagnosis (chi^2^ – 16.19), beginning (chi^2^ – 13.11) and frightened (chi^2^ – 7.21) associated with the TSs evidenced the feelings of apprehension and fright experienced by the participants upon receiving a syphilis diagnosis during pregnancy. The highlighted TSs below reveal the negative emotions experienced by the participants, illustrating their initial reactions to the diagnosis of GS:

*Receiving a syphilis diagnosis was awful, it was unpleasant, it was bad.* [...] *I was also quite scared, I had never had syphilis and didn’t know what it was (M4).*

[...] *Receiving a syphilis diagnosis was tough* [...] *because I didn’t accept it at first,* [...] *for me it was kind of disturbing, frightening (M5).*

Other TSs analyzed revealed that the participants had no prior knowledge about their condition of being infected, nor did they understand what syphilis was, demonstrating the presence of individual vulnerability.

*When I was diagnosed, I didn’t know what it was.* [...] *I found out after they talked to me (M3).*

*When I received the syphilis diagnosis, I didn’t understand.* [...] *I did some research and that’s when I understood (M18).*

### Social Vulnerability

#### Class 4 – Unfavorable Social Determinants Affecting Adherence to Prenatal Care

Class 4 consisted of 13 TSs, representing 20.31% of the material classified for analysis. The lexicons prenatal (chi^2^ – 17.44) and follow-up (chi^2^ – 10.23) associated with the most significant TSs present content that points to the difficulties women reported in continuing prenatal care, such as living far away, understanding the importance of prenatal care in the face of a GS diagnosis, and the lack of a support network, highlighting situations of social vulnerability.

*During my prenatal care, I didn’t receive better care because I stopped attending appointments.* [...] *They came to visit me and they told me to come back for the appointments,* [...] *But I didn’t go because it was too far, and it was very difficult to get there (M10).*

*At the time I was very young, I didn’t care much.* [...] *I went to the first appointment when I found out the pregnancy, and then I stayed away for quite a while. I had no help from anyone at home (M 20).*

### Programmatic Vulnerability

#### Class 4 – Monitoring of GS: Perception of a “Normal” Prenatal Period

Still in Class 4, through the lexicon normal (chi^2^ – 25.97), the perception of prenatal care as normal emerged, even in high-risk situations like GS. This classification is possibly based on a lack of knowledge about the problems experienced and their consequences, since, as the TSs point out, they did not have information about the GS;

*My prenatal care was normal.* [...] *It was my first pregnancy (M2).*

*My prenatal care was normal.* [...] *once a month, and then it turned to every two weeks.* [...] *I was followed up and took the medicine they prescribed, but they didn’t explain much (M9).*

#### Class 5 – Gaps Perceived in Prenatal Care Follow-up

Class 5 was composed of 14 TSs, representing 21.88% of the material classified for analysis, and the lexicons with the highest statistical significance associated with this class were: examination (chi^2^ – 48.13), family clinic (chi^2^ – 18.74), and blood (chi^2^ – 14.63). The specific TSs of this class show that women indicated that prenatal care was perceived as minimal, consisting only of medication, demonstrating the presence of programmatic vulnerability:

*They only administered the medication.* [...] *After that, they only ordered a blood test.* [...] *They asked for nothing more and said nothing more (M5).*

*At the health center, they only said I would have to get the vaccine, but they didn’t tell me how it worked, what was going to happen* [...] *and it was just a blood test (M18).*

Reinforcing the presence of programmatic vulnerability, other TSs from Class 5 also presented perceptions of the care received, specifically in the Family Clinics where they had prenatal consultations, highlighting weaknesses regarding the structure, embracement, organization, bonding, and quality of the health services provided.

*I didn’t get treatment for syphilis at the family clinic because my test at the beginning of my pregnancy was negative and the test results at the end of the pregnancy were inconclusive. Very disorganized! (M12)*

*I didn’t like it, I didn’t receive any guidance on syphilis during pregnancy, how to prevent it and how to transmit it to the fetus (M18).*

#### Class 5 – Syphilis Diagnosis via CS: Unfavorable Outcome

Given the perceived weaknesses in prenatal care, some TSs demonstrate unfavorable outcomes, such as the discovery of syphilis only at the maternity ward, at the child’s birth, that is, the finding of CS. Some women have noticed flaws related to GS, as TSs in class 4 indicate:

*Syphilis was not detected in tests from 5 weeks until my 32nd week.* [...] *I was accompanied, in fact it was not detected at the family clinic, it was only detected at the hospital. My son was already in the ICU with syphilis (M16).*

*I felt my prenatal care wasn’t good, otherwise I wouldn’t have lost my son to syphilis. There at the clinic, the doctor tried to listen to the baby’s heartbeat with three different devices but was unable to hear it. I thought* s*he had to send me to the hospital, somewhere, but she said it was normal.*

## DISCUSSION

Based on the information collected about the perception of GS, the aim was to analyze the susceptibility to this infection and to the disease process from the perspective of vulnerabilities, which result from the interaction of individual, collective, and contextual factors^(1)^. Thus, the concept of vulnerability transcends the biological dimension and incorporates social, economic, cultural, and programmatic determinants, providing a fundamental theoretical basis for integrative and equitable health policies and practices^(20)^.

The results of this study reinforce that syphilis affects women of different age groups, with a particular prevalence among black women, those with low levels of education, and those with lower purchasing power. This profile reflects social vulnerabilities marked by structural racism and inequalities in access to health services. These findings corroborate previous evidence indicating greater exposure of this profile of women to sexually transmitted infections (STIs), due to socioeconomic and cultural factors^(21,22)^. The participants’ low educational level and precarious economic conditions emerge as determinants of barriers to prenatal care and adherence to syphilis treatment, consistent with results from other studies^(23,24)^. These factors are exacerbated by inadequate prenatal care and a lack of preventive and therapeutic guidance, reinforcing the need for integrated strategies that consider the cultural and social specificities of women in more vulnerable contexts^(25)^, as is the case with the participants in this study.

Syphilis, as an STI, presents significant challenges during pregnancy, generating biological, emotional, and social stresses. In the field of health, vulnerability is understood as a human condition that emerges from the dynamic interaction between the individual and the social context, permeated by power relations. This situation becomes critical when there is precariousness, characterized by the absence of individual or collective empowerment. In this sense, precariousness manifests itself in psychological, physical, social, or relational problems. Conversely, empowerment is related to strengthening support networks, reflective practices, spirituality, positive feelings, health promotion, and resilience^(9)^, practices that were neither reported nor experienced by the participants, reinforcing situations of individual and social vulnerability.

Upon receiving a diagnosis of GS, the participants expressed unexpected feelings: surprise, apprehension, and anxiety, often intensified by a lack of knowledge about this infection and its implications for maternal and fetal health. This result highlights the individual vulnerability related to negative feelings experienced when faced with something unknown and obscure^(20)^, which compromises full adherence to the treatment process, as also described in another study^(23)^.

Misinformation about syphilis has been identified as a significant barrier to the prevention and management of the infection, negatively impacting the start of prenatal care and, consequently, early diagnosis. Studies conducted in Brazil and internationally underscore the correlation among low educational levels, lack of knowledge and access to reliable information about syphilis, and lack of connection with healthcare professionals as primary factors contributing to the high incidence and prevalence of this STI^(25,26)^.

In this regard, participatory and more integrated educational actions emerge as fundamental strategies for strengthening selfcare and reducing vulnerabilities^(16,26)^. These findings corroborate a study conducted in the United States that points to the need for expanding testing and treatment of pregnant women and their partners, improving access to and linkage to effective maternal and child care, but above all, strengthening health education initiatives^(27)^.

Gender inequality is also one of the major factors contributing to women’s social vulnerability to STIs, including GS. Gender issues, which place women in a position of inferiority and submission in relation to men, deprive them of decisionmaking power, such as regarding the use of condoms during sexual activity and the treatment of syphilis^(24)^, as can be seen in this study, in which only 12 sexual partners adhered to the treatment. Issues such as financial dependence, difficulty in communicating with partners, fear of domestic violence, and the normalization of male domination prevent women from taking a leading role in their sexual and reproductive health, hindering the prevention of GS. In this context, a scientific, welcoming, non-judgmental, and humanized approach by professionals must consider the intersection of biological, social, and cultural factors to understand gender inequality in the context of GS, highlighting the need for actions that promote women’s empowerment^(28)^.

The interviewees’ perception of prenatal care showed significant weaknesses, mainly regarding its management, which was understood as inadequate, disorganized, and disproportionate to the risks associated with GS. They emphasize that this topic was only addressed in a medicalized and fragmented way, only after the diagnosis was confirmed, thus perpetuating the lack of knowledge about this STI and hindering preventive decision-making, reinforcing situations of vulnerability and, consequently, leading to unfavorable outcomes.

These limitations highlighted programmatic vulnerabilities in health services, characterized by the absence of effective guidelines and appropriate conduct. The gap in the bond between healthcare professionals and pregnant women reflects the need to improve the quality of prenatal care, promoting guidance on prevention, diagnosis, and treatment, in a continuous manner and with follow-up^(29)^. Programmatic vulnerability refers to investments in preventive information and education programs and actions, as well as access to high-quality social and health services that are democratically designed, regularly reviewed and evaluated, and free from institutional discrimination^(12)^.

While prenatal care coverage in Brazil is high, it presents regional inequalities and significant limitations in the quality of care, compromising the early detection and clinical management of GS, perpetuating worrying rates of CS^(28,29)^. The results of this study show that, in many situations, the diagnosis of GS only occurs at the time of delivery, revealing critical shortcomings in prenatal care, where attention is still predominantly focused on performing laboratory tests and prescribing medication, without ensuring comprehensive and effective care. This finding reinforces the need to rethink clinical practice, prioritizing not only the early identification of the problem, but also the continuous follow-up of pregnant women and their partners.

The effectiveness of prenatal care depends on structural and programmatic efforts that overcome social, political, cultural, and economic inequalities, which can directly influence women’s vulnerability to GS^(20,24)^. In this context, PHC services should be reoriented to integrate care strategies that consider not only the clinical picture, but also the social and individual conditions of pregnant women, such as low socioeconomic status, difficulty accessing information and treatments, lack of a support network, and other barriers that increase the risk of vertical transmission of the infection^(22,29)^.

However, this study was developed in a territory historically marked by profound social inequalities, a condition that justifies its choice as an empirical field in accordance with the objective investigated. When analyzing the perception of women diagnosed with GS regarding prenatal care, it becomes essential to understand this phenomenon in contexts where individual, social, and programmatic vulnerabilities manifest themselves more intensely, directly impacting access to health services, the quality of care provided, and maternal and infant outcomes.

Prenatal care should go beyond simply requesting and evaluating screening and diagnostic tests for syphilis or monitoring treatment. It is essential that healthcare professionals assume an educational role with pregnant women and their families, based on embracement and continuous monitoring, offering support and guidance on the risks of infection to maternal and fetal health. Furthermore, strengthening the bonds among the healthcare team, the pregnant woman, and her family generates positive repercussions, such as greater adherence to the proposed therapeutic plan. This relationship of trust fosters regular appointments and allows for more effective follow-up, with systematic and organized monitoring, positively impacting pregnancy progression^(29)^. These are aspects that were not perceived in the statements of the participants in this study, reinforcing the presence of programmatic vulnerability.

Given the presence of vulnerable situations, continuing education for healthcare professionals is essential to improve the management of all aspects of GS beyond diagnosis and treatment. Low-tech and practical educational approaches in healthcare, such as courses and training to qualify healthcare staff, the development of visual tools detailing the flow of diagnosis, treatment, and follow-up at all levels of healthcare, territorial educational workshops with culturally sensitive, updated, and adapted pedagogical resources, the use of Care Pathways and integrated information systems; agreements between services and strengthening of multidisciplinary teams, as well as strategies involving the community and affective-sexual partnerships, are fundamental actions to minimize the vulnerabilities of women diagnosed with GS and promote quality care for this population^(29)^.

One limitation of the study is its reliance on retrospective reports, which are subject to memory biases, and its focus on a single *AP*, making it impossible to generalize the results. However, the research offers a profound and unique understanding of the experiences of the women interviewed, allowing essential reflections for improving health policies and practices in the fight against GS and CS issues.

## CONCLUSION

The study highlights the complexity of GS and the gaps in the management of this condition in PHC services. Individual, social, and programmatic vulnerabilities faced by women affected by GS stand out, as they have been shown to compromise both early detection and timely treatment during prenatal care, resulting in vertical transmission of the disease.

This situation underscores the urgency of a humanized prenatal care, with respectful embracement and continuous and rigorous monitoring, which strengthens the bond between pregnant women and health professionals. It is essential that this care understands the women’s sociocultural and economic context, promoting integrated actions that overcome the vulnerabilities identified.

Strengthening PHC services with low-tech approaches and educational initiatives, coupled with the humanization of care, is urgent and necessary to reduce the rates of GS and, consequently, of CS, thereby promoting the quality of sexual and reproductive health.

## Data Availability

The entire dataset supporting the results of this study was published in the article itself.
