# Supplementary material for: Gestational syphilis and prenatal care: a study in light of individual, social, and programmatic vulnerabilities
Source: Rev Esc Enferm USP. 2026 May 29;60:e20250270. doi: 10.1590/1980-220X-REEUSP-2025-0270en (PMC13220897; doi:10.1590/1980-220X-REEUSP-2025-0270en)
Supplement: Translation

# Translation: Sífilis gestacional e acompanhamento pré-natal: um estudo frente às vulnerabilidades individuais, sociais e programáticas

**DOI:** 10.1590/1980-220X-REEUSP-2025-0270pt

**Authors:** Adriana de Oliveira Sarefino, Ana Beatriz Azevedo Queiroz, Karla Gualberto Silva, Norma Valéria Dantas de Oliveira Souza, Natalia Moreira Leitão Titara, Ana Luiza de Oliveira Carvalho, Juliana da Fonsêca Bezerra, Ana Kedma Correa Pinheiro, Aline Furtado da Rosa

**Affiliations:** 1 Universidade Federal do Rio de Janeiro, Escola de Enfermagem Anna Nery, Rio de Janeiro, RJ, Brasil.

**Keywords:** Sífilis, Infecções Sexualmente Transmissíveis, Vulnerabilidade em Saúde, Atenção Primária à Saúde

## Abstract

**Objetivo:**

Analisar a percepção de mulheres diagnosticadas com sífilis gestacional sobre o acompanhamento pré-natal na Estratégia Saúde da Família e discutir as vulnerabilidades individual, social e programática relacionadas a esse contexto.

**Método:**

Pesquisa qualitativa e descritiva, realizada com 20 mulheres com diagnóstico de sífilis gestacional, notificadas em duas Clínicas da Família localizadas no bairro Santa Cruz, Rio de Janeiro/Brasil. Como coleta de dados foi realizada entrevista semiestruturada individual nas residências das participantes e tratamento dos dados por meio do software IRAMUTEQ.

**Resultados:**

As participantes eram majoritariamente pardas ou pretas, com baixa escolaridade, desemprego e baixa renda, indicando vulnerabilidade social. O diagnóstico de sífilis ocorreu principalmente durante a gestação, sendo alguns casos identificados apenas no parto ou pós-parto. Os resultados apontaram vulnerabilidades individuais, sociais e programáticas, relacionadas à falta de conhecimento, barreiras de acesso e falhas no pré-natal.

**Conclusão:**

A sífilis gestacional está associada a fatores sociais e estruturais que exigem ações integradas de educação em saúde, fortalecimento do pré-natal e ampliação do envolvimento das parcerias no tratamento.

## INTRODUÇÃO

A Sífilis Gestacional (SG) representa um importante desafio para a saúde pública global, especialmente em países subdesenvolvidos ou em desenvolvimento, como ocorre em diversas nações das Américas. A identificação precoce da SG e a implementação de protocolos clínicos durante o pré-natal, parto e puerpério são essenciais para reduzir a incidência das infecções congênitas pela SG^(1)^. A sífilis congênita (SC) pode causar malformações e alterações ósseas, entre outras complicações e manifestações precoces e tardias, como aborto espontâneo, parto prematuro, morte neonatal, baixo peso ao nascer, comprometimento neurológico, incluindo deficiência mental e deficiência auditiva^(2)^.

Nas Américas, o percentual de gestantes com sífilis aumentou 28% nos últimos dois anos, o que resultou no crescimento proporcional dos casos de Sífilis Congênita (SC), alcançando uma estimativa de 4,98 casos por 1.000 nascidos vivos em 2022, valor que supera significativamente a meta estabelecida pela Organização Mundial da Saúde (OMS) de 0,5 caso por 1.000 nascidos vivos^(1)^. Ainda segundo a OMS^(3)^, anualmente, a SG é responsável por mais de 300 mil mortes fetais e neonatais no mundo, além de colocar em risco de morte prematura mais de 200 mil crianças. No Brasil, os casos de sífilis adquirida, SG e SC apresentaram aumento significativo nos últimos anos, evidenciando a necessidade de intervenções eficazes e abrangentes no cuidado pré-natal, levando em consideração as situações de vulnerabilidades^(2)^.

A assistência pré-natal corresponde à melhor oportunidade de rastreamento, diagnóstico, tratamento e prevenção da SG, tanto para as gestantes, quanto para suas parcerias. O diagnóstico precoce e o tratamento oportuno da sífilis em gestantes são etapas cruciais para a redução da transmissão vertical e para a prevenção de complicações. Recomenda-se que as gestantes sejam testadas pelo menos na primeira consulta de pré-natal, preferencialmente, no primeiro trimestre de gestação, no início do terceiro trimestre e no momento da internação para o parto. Quando diagnosticadas, devem ser tratadas imediatamente, e a continuidade do cuidado deve ser assegurada pela equipe de saúde^(2)^.

No Brasil, a Estratégia Saúde da Família (ESF), no contexto da Atenção Primária à Saúde (APS), desempenha um papel fundamental na ampliação do acesso e na promoção de cuidados pré-natais de qualidade, sendo considerada uma estratégia essencial para o controle e a prevenção da SG e SC^(4)^.

Embora o acesso ao pré-natal seja amplamente garantido no Brasil, estudo aponta lacunas na qualidade do atendimento oferecido, o que gera preocupantes situações de vulnerabilidades^(5)^. Pesquisa realizada no Rio Grande do Sul evidenciou que, mesmo entre gestantes que realizaram o pré-natal, a inadequação do tratamento (52%) e a ausência desses (32,4%) permanecem como um dos fatores de vulnerabilidades à SC, assim como o não tratamento das parcerias sexuais, que é um dos principais obstáculos para o controle efetivo da sífilis^(6)^.

A ausência de acesso a serviços de saúde de qualidade, especialmente no que se refere à realização de triagem e intervenções terapêuticas efetivas, permanece como um fator significativo de vulnerabilidade para a elevada prevalência dessa condição em diversas regiões do mundo^(7)^.

Ainda que a SG não figure entre as 20 doenças tropicais negligenciadas, ela integra as estratégias e metas estabelecidas pela OMS^(8)^ para a eliminação da negligência e o alcance dos Objetivos de Desenvolvimento Sustentável (ODS) até 2030. A literatura científica internacional, contudo, reconhece a SG como uma doença negligenciada^(9)^, fortemente associada a condições de vulnerabilidade que afetam populações específicas^(10,11)^.

É importante ressaltar que a definição de vulnerabilidade relaciona-se com um conjunto de aspectos individuais, coletivos e contextuais que ocasionam maior suscetibilidade à infecção e ao adoecimento, sendo indissociável, em níveis maiores ou menores, do acesso a recursos para assegurar sua proteção^(12)^.

A vulnerabilidade em saúde é compreendida como uma condição humana, decorrente da interatividade entre o sujeito e o social, a partir das relações de poder, e que pode estar em situação de precariedade, quando o empoderamento do sujeito ou do coletivo não está sendo vivenciado no momento^(13)^. Estas fragilidades envolvem aspectos sociodemográficos, estando entre eles o acesso aos serviços de saúde, as condições socioeconômicas e a dimensão cultural, que são oriundos da própria sociedade e interferem na movimentação dos indivíduos e grupos dentro de uma hierarquia social^(11)^.

Estudos nacionais e internacionais evidenciam que determinantes sociais desfavoráveis, como baixa escolaridade, renda reduzida e migração, bem como o déficit na educação em saúde, contribuem para maior condição de vulnerabilidade da SG e seus desdobramentos^(14,15)^. Nesse contexto, a análise das vulnerabilidades torna-se fundamental para compreender os fatores que influenciam as **altas taxas de incidência e prevalência**, a persistência da SG e a ocorrência da transmissão vertical. Essas vulnerabilidades podem ser compreendidas a partir de três dimensões interdependentes: a individual, que envolve aspectos biológicos, comportamentais e emocionais; a social, relacionada ao contexto e às relações interpessoais; e a programática, que diz respeito às políticas públicas, programas e serviços de saúde disponíveis^(16)^.

Essas dimensões englobam aspectos individuais relacionados à exposição e à suscetibilidade ao agravo em questão; características contextuais e dos vínculos sociais estabelecidos; bem como o modo como as tecnologias expressas em políticas, programas, serviços e ações operam nesses contextos e influenciam as situações^(16)^. Desse modo, essas três dimensões analíticas contribuem para compreender a relação entre o indivíduo e a vulnerabilidade.

Para abordar efetivamente a SG é crucial a identificação dos fatores relacionados às vulnerabilidades que favorecem a persistência da SG e implementar estratégias de saúde pública que considerem as vulnerabilidades e promovam o acesso equitativo aos serviços de saúde. Para tanto, faz-se necessária uma estrutura de análise que incorpore o comportamento individual, o âmbito coletivo e o social^(16)^.

Diante dessa problemática, surge o questionamento: qual a percepção das mulheres diagnosticadas com SG sobre o acompanhamento pré-natal realizado na ESF e as possíveis vulnerabilidades individuais, sociais e programáticas associadas a esse acompanhamento?

A relevância deste estudo justifica-se pelo impacto da SC no cenário brasileiro, considerando as consequências imediatas e de longo prazo para os recém-nascidos e a saúde neonatal. Nesse contexto, o presente estudo tem como objetivo analisar a percepção de mulheres diagnosticadas com SG sobre o acompanhamento pré-natal na ESF e discutir as vulnerabilidades individual, social e programática.

## MÉTODO

### Tipo de Estudo

Trata-se de uma pesquisa descritiva, de abordagem qualitativa, orientada pelas diretrizes do *Consolidated Criteria for Reporting Qualitative Research* (COREQ)^(17)^.

### Local, População e Critérios de Seleção

O campo de pesquisa foi conduzido na Área de Planejamento (AP) 5.3, localizada na zona oeste do município do Rio de Janeiro/Brasil, nas residências de mulheres notificadas com SG em duas Clínicas da Família, localizadas no bairro Santa Cruz. Ressalta-se que a escolha dessa área para a pesquisa justifica-se pelo fato de ser uma área com alta incidência de casos de SC, de acordo com dados extraídos do Sistema de Informação de Agravos de Notificação (SINAN). A pesquisa foi conduzida exclusivamente pela pesquisadora, enfermeira atuante na APS e conhecedora do território estudado, o que favoreceu a aproximação com as participantes e a compreensão das vivências relatadas. O contato com as participantes ocorreu de forma ética, empática e respeitosa, assegurando a confidencialidade das informações e a neutralidade analítica na interpretação dos resultados.

Participaram do estudo 20 mulheres que tiveram o diagnóstico de SG, atendendo aos critérios de inclusão: idade mínima de 18 anos, que tiveram acompanhamento pré-natal na ESF da AP 5.3 e notificações de SG entre os anos de 2016 e 2021. Este período corresponde à implementação da Agenda de Ações Estratégicas para Redução da Sífilis no Brasil, que priorizou a integração de instituições de ensino e pesquisa para ampliar as estratégias de enfrentamento ao agravo^(18)^. Foram excluídas mulheres notificadas no SINAN que não residiam no território do estudo.

### Coleta de Dados

A coleta de dados ocorreu entre agosto de 2023 e março de 2024 e a seleção das participantes deu-se a partir da tabela de casos notificados fornecidos pela Divisão de Vigilância em Saúde da coordenadoria de APS da AP 5.3. A referida tabela continha um total de 67 mulheres e algumas informações como idade, endereço e telefone. Desse total, 19 não atendiam aos critérios de inclusão, 13 não moravam mais no endereço informado, quatro não quiseram participar da pesquisa por motivos pessoais e 11 não atenderam as ligações telefônicas. Assim, com 20 participantes foram agendadas as visitas domiciliares, conforme preferência de dia e horários das mulheres, e a coleta de dados foi realizada nas residências das participantes, em cômodos individualizados para garantir a privacidade das informações, com presença apenas da pesquisadora.

A técnica de coleta de dados utilizada foi um formulário com perguntas fechadas para traçar a caracterização sociodemográfica e de dados referente ao diagnóstico da SG. O instrumento continha os seguintes dados: idade, escolaridade, raça/cor autodeclarada, religião, profissão/ocupação, renda familiar, benefícios sociais. Quanto ao diagnóstico da SG, os dados coletados foram: idade gestacional do início do pré-natal, número de consultas, período do diagnóstico da sífilis, tratamento realizado e tratamento das parcerias sexuais.

A segunda etapa de coleta de dados foi uma entrevista semiestruturada individual em profundidade contendo os seguintes tópicos: percepção sobre o acompanhamento prénatal após o diagnóstico da sífilis na gestação, como foi ter recebido o diagnóstico, como foram os esclarecimentos/orientações em relação à SG e percepção quanto ao acesso aos serviços de saúde e acompanhamento em relação ao diagnóstico, tratamento da SG e prevenção da SC. Ressalta-se que foram realizadas duas entrevistas pilotos com gestantes diagnosticadas com SC fora do critério de inclusão do recorte temporal, no intuito de ajustes no roteiro de entrevista, mas que não foram contabilizadas no total da amostra.

As entrevistas foram gravadas, após a leitura e assinatura do Termo de Consentimento Livre e Esclarecido, mediante autorização, com o uso de aplicativo em dispositivo móvel, tendo uma duração média de 30 minutos e, posteriormente, transcritas na íntegra para análise.

### Análise e Tratamento dos Dados

O processo de análise de dados das entrevistas foi realizado utilizando o software IRAMUTEQ (*Interface de R pour les Analyses Multidimensionnelles de Textes et de Questionnaires*), por meio de análise de conteúdo lexical. Esse software permite integrar métodos estatísticos e análises subjetivas, com representações gráficas de fácil interpretação e aplicabilidade em bases de dados extensas, desde que o corpus textual seja devidamente preparado^(19)^. O corpus textual foi organizado, compreendendo os conceitos de corpus, texto e segmento de texto (ST). O corpus abarcou o conjunto das 20 entrevistas, cada uma tratada como texto, sendo o ST definido como unidade analítica com tamanho aproximado de três linhas, ajustado automaticamente pelo IRAMUTEQ^(19)^. As linhas de comando, precedidas por quatro asteriscos (****) e seguidas por códigos específicos, identificaram as participantes utilizando a letra M de mulher seguida do numeral (M_1 a M_20) e as variáveis estudadas foram idade, possui parceiro, realizou tratamento e escolaridade. Todas essas etapas asseguraram o rigor metodológico, o anonimato das participantes e a organização necessária para o processamento e análise dos dados.

Este estudo utilizou a Análise por Classificação Hierárquica Descendente (CHD), que agrupa ST com base em vocabulários semelhantes, fragmentando o corpus textual de acordo com a frequência de formas reduzidas^(19)^. O objetivo principal é identificar classes de vocabulário que apresentem homogeneidade interna e heterogeneidade entre si, permitindo a análise detalhada de ST por meio de testes de Qui-quadrado (chi^2^) para calcular distâncias e proximidades entre os termos^(19)^.

A análise da CHD dividiu o corpus textual em cinco classes temáticas, totalizando 81,01% de aproveitamento do material analisado. Essa repartição revelou dois blocos temáticos, sendo o bloco1 denominado de “A sífilis gestacional e o acompanhamento pré-natal: a presença das vulnerabilidades”, sendo formado pelas classes 1, 4 e 5. Este bloco foi o que respondeu ao objetivo deste estudo, abordando os aspectos relacionados à identificação da sífilis durante a gestação, a percepção das participantes sobre os atendimentos recebidos e as vulnerabilidades individuais, programáticas e sociais no acompanhamento pré-natal.

Os resultados brutos fornecidos pelo software foram tratados e interpretados, buscando atribuir significados e validade às classes encontradas. A análise incluiu deduções lógicas, descrição e contextualização com base em literatura científica relevante, permitindo uma interpretação mais aprofundada dos discursos e uma compreensão das mensagens implícitas nos vocabulários identificados, que foram analisadas e nomeadas com base nos referenciais teóricos sobre a SG e dimensões das vulnerabilidades.

### Aspectos Éticos

O estudo foi conduzido em conformidade com a Resolução nº 466/2012 do Conselho Nacional de Saúde/MS, que regula pesquisas envolvendo seres humanos, e com a Resolução nº 580/2018, aplicável a pesquisas realizadas em instituições do Sistema Único de Saúde (SUS). Foi apreciado e aprovado pelo Comitê de Ética em Pesquisa da Universidade Federal do Rio de Janeiro – Escola de Enfermagem Anna Nery/ Hospital Escola São Francisco de Assis, CAAE 66485023.2.0000.5238 e número do parecer 6.175.541, e pelo Comitê de Ética em Pesquisa da Secretaria Municipal de Saúde do Rio de Janeiro, CAAE 66485023.2.3001.5279 e número do parecer 6.210.457.

## RESULTADOS

As participantes deste estudo foram 20 mulheres, com idades entre 20 e 48 anos, sendo a faixa etária de 30 a 39 anos a mais prevalente (nove mulheres). Em relação à autodeclaração de raça/cor, 13 identificaram-se como pardas e cinco como pretas, totalizando 18 mulheres negras. A escolaridade foi predominantemente baixa, com oito mulheres apresentando o ensino médio incompleto. Sobre a religião, 12 declararam não possuir vínculo religioso, enquanto oito afirmaram praticar religiões cristãs, sendo seis evangélicas e duas católicas. No que se refere à conjugalidade, nove eram solteiras sem parcerias, enquanto 11 conviviam com companheiros. Entre as solteiras, sete viviam apenas com os filhos, enquanto as mulheres com companheiros residiam, majoritariamente, com marido e filhos. A análise da ocupação revelou que 14 estavam desempregadas e seis exerciam atividades remuneradas. A renda familiar variou entre menos de um e quatro salários mínimos, sendo que 10 sobreviviam com menos de um salário mínimo. Ademais, 13 recebiam algum benefício social do governo.

No acompanhamento pré-natal, 12 iniciaram antes das 12 semanas de gestação, sendo que seis não recordavam a idade gestacional do início. O número de consultas variou de quatro a 14, com nove mulheres realizando menos de seis consultas, número inferior ao recomendado pelo Ministério da Saúde^(2).^ Adicionalmente, o diagnóstico de sífilis ocorreu majoritariamente durante a gestação em 15 mulheres, enquanto cinco foram diagnosticadas somente no momento do parto ou após o nascimento do filho. Entre as gestantes diagnosticadas, 15 realizaram tratamento com Benzilpenicilina, duas usaram outras medicações devido a alergias e uma não recebeu tratamento. No que diz respeito às parcerias sexuais, 12 afirmaram que a parceria realizou o tratamento, enquanto 8 participantes declararam que os parceiros não aceitaram fazer uso da medicação.

Nos resultados das entrevistas, referente à análise da CHD do software IRAMUTEQ, para este artigo, foram analisadas as classes correspondentes ao bloco 1, classes 1, 4 e 5, e organizadas conforme as vulnerabilidades apresentadas.

Dessa forma, as classes geradas pelo IRAMUTEQ foram interpretadas à luz das três dimensões da vulnerabilidade em saúde: individual, social e programática. Assim, uma mesma classe pode conter conteúdos que dialogam com mais de uma dimensão, sendo apresentada conforme o enfoque analítico predominante em cada eixo.

### Vulnerabilidade Individual

#### Classe 1 – Diagnóstico da Sífilis Gestacional e a (Des) Informação: o Inesperado da Revelação

A Classe 1 foi composta por 9 ST, representando 14,06% do material classificado para análise. Os léxicos diagnóstico (chi^2^ – 16.19), início (chi^2^ – 13.11) e assustada (chi^2^ – 7.21) associados aos ST evidenciaram os sentimentos de apreensão e susto vivenciados pelas participantes ao receberem o diagnóstico de sífilis durante a gestação. Os ST destacados a seguir revelam as emoções negativas experimentadas pelas participantes, ilustrando as reações iniciais frente ao diagnóstico de SG:

*Receber o diagnóstico da sífilis foi horrível, foi chato, ruim* [...], *eu fiquei também bem assustada, nunca tinha tido sífilis e não sabia o que era (M4).*

[...] *receber o diagnóstico da sífilis foi difícil* [...] *porque eu não aceitava de início,* [...] *para mim foi meio perturbador, assustador (M5).*

Outros ST analisados revelaram que as participantes não possuíam conhecimento prévio sobre sua condição de infectadas, tampouco compreendiam o que era a sífilis, demonstrando a presença da vulnerabilidade individual.

*Quando eu fui diagnosticada eu não sabia o que era,* [...] *eu fiquei sabendo depois que eles conversaram comigo (M3).*

*Quando recebi o diagnóstico da sífilis eu não entendia,* [...] *fui pesquisar e foi que eu fui entender (M18).*

### Vulnerabilidade Social

#### Classe 4 – Determinantes Sociais Desfavoráveis que Afetam a Adesão ao Acompanhamento Pré-natal

A Classe 4 foi composta por 13 ST, representando 20,31% do material analisado. Os léxicos pré-natal (chi^2^ – 17.44) e acompanhamento (chi^2^ – 10.23) associados aos ST de maior significância apresentam conteúdos que apontam as dificuldades que as mulheres relataram em dar continuidade ao acompanhamento pré-natal, como morar longe, entender a importância do pré-natal frente ao diagnóstico da SG e a falta de uma rede de apoio, emergindo situações de vulnerabilidades sociais.

*No meu acompanhamento pré-natal não fui atendida melhor porque eu não fui mais às consultas,* [...] *vieram me visitar e falaram para eu voltar para as consultas,* [...] *mas eu não fui porque era muito longe, tinha muita dificuldade de pegar condução (M10).*

*Na época eu era muito nova, não estava me importando muito,* [...] *fui à primeira consulta quando eu descobri a gravidez, e depois eu fiquei um bom tempo sem ir. Não tinha ajuda de ninguém em casa (M 20).*

### Vulnerabilidade Programática

#### Classe 4 – Acompanhamento à SG: Percepção de um Pré-natal “Normal”

Ainda na Classe 4, por meio do léxico normal (chi^2^ – 25.97), emergiu a percepção do acompanhamento pré-natal como comum, mesmo diante de um quadro de risco como a SG. Essa classificação, possivelmente, tem bases no desconhecimento sobre a problemática vivenciada e suas consequências, pois como destacam os ST, não tiveram informação sobre a SG;

*Meu acompanhamento pré-natal foi normal,* [...] *foi minha primeira gravidez (M2).*

*Meu acompanhamento pré-natal foi normal,* [...] *uma vez por mês e depois já era de quinze em quinze dias *[...]*eu fiz o acompanhamento e tomei o remédio que eles mandaram, mas não explicavam muita coisa (M9).*

#### Classe 5 – Lacunas Percebidas no Acompanhamento do Pré-natal

A Classe 5 foi composta por 14 ST, representando 21,88% do material classificado para análise e os léxicos com maior significância estatística associados a essa classe foram: exame (chi^2^ – 48.13), clínica da família (chi^2^ – 18.74) e sangue (chi^2^ – 14.63). Os ST específicos desta classe mostram que as mulheres apontaram que o acompanhamento pré-natal foi percebido como um atendimento mínimo, apenas medicamentoso, demonstrando a presença da vulnerabilidade programática:

*Eles só fizeram a medicação,* [...] *depois só pediram exame de sangue,* [...] *não pediram mais nada e não falaram mais nada (M5).*

*Nos serviços de saúde eles só falaram que eu teria que tomar a vacina, mas não me falaram como é que era, o que ia acontecer *[...]* e foi só exame de sangue (M18).*

Reforçando a presença da vulnerabilidade programática, outros ST da Classe 5 também apresentaram percepções do atendimento recebido, especificamente nas Clínicas da Família onde realizaram as consultas de pré-natal, evidenciando as fragilidades quanto a estrutura, acolhimento, organização, vínculo e qualidade dos serviços de saúde ofertados.

*Não fiz o tratamento para sífilis na clínica da família porque meu exame do início da gestação deu negativo e no final da gravidez deu exame duvidoso. Muito desorganizado! (M12)*

*Não gostei, não recebi orientação nenhuma sobre a sífilis na gestação, como prevenir e transmitir para o feto (M18).*

#### Classe 5 – Descoberta da Sífilis por Meio da SC: Desfecho Desfavorável

Frente às fragilidades percebidas no acompanhamento pré- natal, alguns ST demonstram o desfecho desfavorável, como a descoberta da sífilis apenas na maternidade, no nascimento do filho, ou seja, a constatação da SC. Algumas mulheres perceberam falhas com relação a SG, conforme os ST na classe 4, apontam:

*A sífilis não foi detectada nos exames de 5 semanas até as minhas 32 semanas.* [...] *Fui acompanhada, sendo que não tinha sido detectada na clínica da família, só foi detectada no hospital. Já com meu filho na UTI com sífilis (M16).*

*Meu acompanhamento pré-natal achei que não foi bom, senão eu não teria perdido meu filho com sífilis. Lá na Clínica, a médica tentou escutar o coração do bebê com três aparelhos e não conseguiu escutar. Eu achava que ela tinha que me mandar para o hospital, para algum lugar, mas ela falou que era normal.*

## DISCUSSÃO

Com base nas informações coletadas sobre a percepção da SG, buscou-se analisar sob a perspectiva das vulnerabilidades, que resultam da interação de fatores individuais, coletivos e contextuais^(1)^, a suscetibilidade à essa infecção e ao processo de adoecimento. Dessa forma, o conceito de vulnerabilidade transcende a dimensão biológica e incorpora determinantes sociais, econômicos, culturais e programáticos, oferecendo uma base teórica fundamental para políticas e práticas de saúde integradoras e equitativas^(20)^.

Os resultados deste estudo reforçam que a sífilis afeta mulheres em diferentes faixas etárias, com destaque para a predominância entre mulheres negras, de baixa escolaridade e com menor poder aquisitivo. Tal perfil reflete vulnerabilidades sociais marcadas pelo racismo estrutural e pelas desigualdades no acesso aos serviços de saúde. Esses achados corroboram evidências anteriores que indicam maior exposição desse perfil de mulheres às infecções sexualmente transmissíveis (IST), em razão de fatores socioeconômicos e culturais^(21,22)^. A baixa escolaridade e as condições econômicas precárias das participantes emergem como determinantes de barreiras ao pré-natal e à adesão ao tratamento da sífilis, em consonância com resultados de outros estudos^(23,24)^. Esses fatores são agravados pela assistência inadequada do pré-natal e pela escassez de orientações preventivas e terapêuticas, o que reforça a necessidade de estratégias integradas que considerem as especificidades culturais e sociais das mulheres em contextos de maior vulnerabilidade^(25)^, como é o caso das participantes desse estudo.

A sífilis, enquanto uma IST, apresenta desafios significativos durante a gestação, gerando tensões biológicas, emocionais e sociais. No campo da saúde, a vulnerabilidade é entendida como uma condição humana que emerge da interação dinâmica entre o sujeito e o contexto social, permeada pelas relações de poder. Essa condição torna-se crítica quando há precariedade, caracterizada pela ausência de empoderamento individual ou coletivo. A precariedade, nesse sentido, manifesta-se em problemas psíquicos, físicos, sociais ou relacionais. Por outro lado, o empoderamento relaciona-se com o fortalecimento de redes de apoio, práticas reflexivas, espiritualidade, sentimentos positivos, promoção da saúde e resiliência^(9)^, práticas essas que não foram relatadas e nem vivenciadas pelas participantes, reforçando as situações de vulnerabilidades individual e social.

Diante do diagnóstico da SG, as participantes expressaram sentimentos inesperados: surpresa, apreensão e ansiedade, frequentemente intensificados pelo desconhecimento sobre essa infecção e suas implicações para saúde materno-fetal. Esse resultado evidencia a vulnerabilidade individual relacionada aos sentimentos negativos vivenciados frente a algo ignorado e obscuro^(20)^, o que compromete a adesão integral ao processo de tratamento, conforme também descrito em outro estudo^(23)^.

A desinformação sobre a sífilis foi identificada como uma barreira relevante para a prevenção e o manejo da infecção, impactando negativamente no início do pré-natal e, consequentemente, no diagnóstico precoce. Estudos realizados no Brasil e internacionalmente destacam a correlação entre baixo nível educacional, falta de conhecimento e acesso às informações confiáveis sobre a sífilis e não vínculo com os profissionais de saúde como fatores primordiais para as altas incidências e prevalências dessa IST^(25,26)^.

Nesse contexto, ações educativas participativas e com maior integração emergem como estratégias fundamentais para o fortalecimento do autocuidado e a redução das vulnerabilidades^(16,26)^. Estes achados corroboram um estudo desenvolvido nos Estados Unidos que aponta para a necessidade da ampliação da testagem e tratamento de gestantes e suas parcerias, melhoria do acesso e da vinculação a cuidados materno-infantis eficazes, mas principalmente, do fortalecimento das ações de educação em saúde^(27)^.

A desigualdade de gênero é, também, um dos fatores de maior vulnerabilidade social das mulheres frente às IST, inclusive a SG. As questões de gênero, que colocam as mulheres em posição de inferioridade e submissão em relação aos homens, privam-nas do poder de decisão, como por exemplo, o uso de preservativos durante as atividades sexuais e ao tratamento da Sífilis^(24)^, como pode ser verificado neste estudo, pois apenas 12 parcerias sexuais aderiram ao tratamento. Questões como dependência financeira, dificuldade em dialogar com as parcerias, o medo da violência doméstica e a naturalização da dominação masculina impedem que as mulheres assumam protagonismo em sua saúde sexual e reprodutiva, dificultando a prevenção da SG. Nessa conjuntura, uma abordagem científica, acolhedora, sem julgamentos e humanizada dos profissionais deve considerar a interseção dos fatores biológicos, sociais e culturais para compreender a desigualdade de gênero no contexto da SG, destacando a necessidade de ações que promovam o empoderamento feminino^(28)^.

A percepção do acompanhamento pré-natal pelas entrevistadas apresentou fragilidades significativas, principalmente com relação à condução, que foi entendida como inadequada, desorganizada e desproporcional aos riscos associados à SG. Destacam que foi uma temática apenas abordada de forma medicalizadora e fragmentada, somente quando confirmado o diagnóstico, perpetuando assim o desconhecimento sobre essa IST e dificultando a tomada de decisões preventivas, reforçando as situações de vulnerabilidades e, consequentemente, trazendo desfechos desfavoráveis.

Essas limitações evidenciaram as vulnerabilidades programáticas nos serviços de saúde, caracterizadas pela ausência de orientações eficazes e condutas apropriadas. Lacuna no vínculo entre os profissionais de saúde e as gestantes reflete a necessidade de melhorar a qualidade da assistência pré-natal, promovendo orientações sobre prevenção, diagnóstico e tratamento, de forma contínua e com acompanhamento^(29)^. A vulnerabilidade programática refere-se aos investimentos em ações e programas de informação e educação preventivas, como também ao acesso a serviços sociais e de saúde, com qualidade elevada, democraticamente traçados, regularmente retroalimentados e avaliados e sem discriminação institucional^(12)^.

A cobertura do pré-natal no Brasil, embora elevada, apresenta desigualdades regionais e limitações significativas na qualidade do atendimento, o que compromete a detecção precoce e o manejo clínico da SG, perpetuando taxas preocupantes de SC^(28,29)^. Os resultados deste estudo evidenciam que, em diversas situações, o diagnóstico da SG ocorre apenas no momento do parto, o que revela falhas críticas no acompanhamento pré-natal, cuja atenção ainda se concentra predominantemente na realização de exames laboratoriais e na prescrição medicamentosa, sem garantir integralidade e efetividade no cuidado. Essa constatação reforça a necessidade de repensar a prática clínica, priorizando não só a identificação precoce do problema, mas o seguimento contínuo das gestantes e suas parcerias.

A efetividade das ações de pré-natal depende de esforços estruturais e programáticos que superem desigualdades sociais, políticas, culturais e econômicas, capazes de influenciar diretamente a vulnerabilidade das mulheres frente à SG^(20,24)^. Nesse contexto, os serviços de APS devem ser reorientados para integrar estratégias de cuidado que considerem não apenas o quadro clínico, mas também as condições sociais e individuais das gestantes, como baixo nível socioeconômico, dificuldade de acesso a informações e tratamentos, ausência de rede de apoio e outras barreiras que aumentam o risco de transmissão vertical da infecção^(22,29)^.

Entretanto, este estudo foi desenvolvido em um território historicamente marcado por profundas desigualdades sociais, condição que justifica sua escolha como campo empírico em consonância com o objetivo investigado. Ao analisar a percepção de mulheres diagnosticadas com SG acerca do acompanhamento pré-natal, torna-se imprescindível compreender esse fenômeno em contextos nos quais as vulnerabilidades individuais, sociais e programáticas manifestam-se de forma mais intensa, incidindo diretamente sobre o acesso aos serviços de saúde, a qualidade do cuidado ofertado e os desfechos materno-infantis.

Os cuidados no pré-natal devem ir além da mera solicitação e avaliação de exames de triagem e diagnóstico da sífilis ou do seguimento do tratamento. É essencial que os profissionais de saúde assumam um papel educativo junto à gestante e sua família, pautado no acolhimento e no acompanhamento contínuo, oferecendo apoio e orientações sobre os riscos da infecção para a saúde materna e fetal. Ademais, o fortalecimento dos vínculos entre a equipe de saúde, a gestante e seus familiares geram repercussões positivas, como maior aderência ao plano terapêutico proposto. Essa relação de confiança favorece a regularidade nas consultas e possibilita um acompanhamento mais efetivo, com monitoramento sistemático e organizado, impactando positivamente na evolução da gestação^(29)^. Estes são aspectos que não foram percebidos nas falas das participantes desse estudo, reforçando a presença da vulnerabilidade programática.

Frente à presença das situações de vulnerabilidade, a educação continuada para profissionais de saúde faz-se essencial para melhorar o manejo de toda a problemática da SG para além do diagnóstico e do tratamento. Tecnologias leves e práticas educativas em saúde, como cursos e treinamentos para qualificar a equipe em saúde, elaboração de ferramentas visuais que detalhem o fluxo de diagnóstico, tratamento e acompanhamento em todos os níveis e atenção de saúde, oficinas educativas territoriais com recursos pedagógicos culturalmente sensíveis, atualizados e adaptados às diversas realidades, o uso das Linhas de Cuidado e sistemas de informação integrados; pactuação entre serviços e fortalecimento de equipes multiprofissionais, assim como, estratégias que envolvam a comunidade e as parcerias afetivo-sexuais são ações fundamentais para minimizar as vulnerabilidades das mulheres diagnosticadas com SG e promover a qualidade do cuidado a essa clientela^(29)^.

Como limitação do estudo destaca-se a dependência de relatos retrospectivos sujeitos a vieses de memória e o enfoque em uma única AP, que impossibilita a generalização dos resultados. Contudo, a investigação oferece uma compreensão aprofundada e singular das experiências das mulheres entrevistadas, possibilitando reflexões essenciais para a melhoria das políticas e práticas de saúde frente ao combate da problemática da SG e SC.

## CONCLUSÃO

O estudo evidencia a complexidade da SG e as lacunas no manejo dessa condição nos serviços de APS. Destacam-se as vulnerabilidades individuais, sociais e programáticas enfrentadas pelas mulheres acometidas com SG, que demonstraram comprometer tanto a detecção precoce quanto o tratamento oportuno durante o pré-natal, resultando na transmissão vertical da doença.

Essa situação ressalta a urgência de um pré-natal humanizado, com acolhimento respeitoso e acompanhamento contínuo e rigoroso, que fortaleça o vínculo entre as gestantes e os profissionais de saúde, sendo fundamental que esse cuidado entenda o contexto sociocultural e econômico das mulheres, promovendo ações integradas que superem as vulnerabilidades apontadas.

O fortalecimento dos serviços de APS com tecnologias leves e ações educativas associados à humanização do cuidado faz-se urgente e necessário visando reduzir os índices de SG e, consequentemente, da SC, promovendo, dessa forma, a qualidade da saúde sexual e reprodutiva.

## Data Availability

Todo o conjunto de dados que dá suporte aos resultados deste estudo foi publicado no próprio artigo.

# Translation: Sífilis gestacional y atención prenatal: un estudio a la luz de las vulnerabilidades individuales, sociales y programáticas

**DOI:** 10.1590/1980-220X-REEUSP-2025-0270es

**Authors:** Adriana de Oliveira Sarefino, Ana Beatriz Azevedo Queiroz, Karla Gualberto Silva, Norma Valéria Dantas de Oliveira Souza, Natalia Moreira Leitão Titara, Ana Luiza de Oliveira Carvalho, Juliana da Fonsêca Bezerra, Ana Kedma Correa Pinheiro, Aline Furtado da Rosa

**Affiliations:** 1 Universidade Federal do Rio de Janeiro, Escola de Enfermagem Anna Nery, Rio de Janeiro, RJ, Brasil.

**Keywords:** Sífilis, Enfermedades de Transmisión Sexual, Vulnerabilidad en Salud, Atención Primaria de Salud

## Abstract

**Objetivo:**

Analizar la percepción de las mujeres diagnosticadas con sífilis gestacional respecto a la atención prenatal en el marco de la Estrategia de Salud Familiar y debatir las vulnerabilidades individuales, sociales y programáticas relacionadas con este contexto.

**Método:**

Investigación cualitativa y descriptiva, realizada con 20 mujeres diagnosticadas con sífilis gestacional, cuyos resultados se obtuvieron en dos clínicas familiares ubicadas en el barrio de Santa Cruz, Río de Janeiro/Brasil. La recopilación de datos se llevó a cabo mediante entrevistas individuales semiestructuradas en los domicilios de las participantes, y el procesamiento de datos se realizó utilizando el software IRAMUTEQ.

**Resultados:**

Las participantes eran en su mayoría mestizas o negras, con bajos niveles de educación, desempleo y bajos ingresos, lo que indica vulnerabilidad social. La sífilis se diagnosticaba principalmente durante el embarazo, aunque algunos casos solo se identificaban durante el parto o el posparto. Los resultados señalaron vulnerabilidades individuales, sociales y programáticas relacionadas con la falta de conocimiento, las barreras de acceso y las deficiencias en la atención prenatal.

**Conclusión:**

La sífilis gestacional está asociada a factores sociales y estructurales que requieren acciones integradas en materia de educación para la salud, fortalecimiento de la atención prenatal y ampliación de la participación de las parejas en el tratamiento.

## INTRODUCCIÓN

La sífilis gestacional (SG) representa un importante desafío para la salud pública mundial, especialmente en países subdesarrollados o en desarrollo, como ocurre en varias naciones de las Américas. La identificación temprana de la SG y la implementación de protocolos clínicos durante la atención prenatal, el parto y el puerperio son esenciales para reducir la incidencia de infecciones congénitas por SG^(1)^. La sífilis congénita (SC) puede causar malformaciones y alteraciones óseas, entre otras complicaciones y manifestaciones tempranas y tardías, como aborto espontáneo, parto prematuro, muerte neonatal, bajo peso al nacer, compromiso neurológico, incluyendo discapacidad intelectual y discapacidad auditiva^(2)^.

En las Américas, el porcentaje de gestantes con sífilis aumentó un 28% en los últimos dos años, lo que dio lugar a un crecimiento proporcional de los casos de sífilis congénita (SC), alcanzando una estimación de 4,98 casos por cada 1.000 nacidos vivos en 2022, cifra que supera significativamente la meta establecida por la Organización Mundial de la Salud (OMS) de 0,5 casos por cada 1.000 nacidos vivos^(1)^. Según la OMS^(3)^, la SG es responsable anualmente de más de 300.000 muertes fetales y neonatales en el mundo, además de poner en riesgo de muerte prematura a más de 200.000 niños. En Brasil, los casos de sífilis adquirida, SG y SC han presentado un aumento significativo en los últimos años, lo que pone de manifiesto la necesidad de intervenciones eficaces y amplias en la atención prenatal, tomando en cuenta las situaciones de vulnerabilidad^(2)^.

La atención prenatal representa la mejor oportunidad para el cribado, el diagnóstico, el tratamiento y la prevención de la SG, tanto para las gestantes como para sus parejas. El diagnóstico precoz y el tratamiento oportuno de la sífilis en gestantes son etapas cruciales para reducir la transmisión vertical y prevenir complicaciones. Se recomienda que las gestantes se sometan a pruebas de detección al menos en la primera consulta prenatal, preferiblemente en el primer trimestre de gestación, al inicio del tercer trimestre y en el momento del ingreso para el parto. Cuando se diagnostica, deben recibir tratamiento de inmediato, y el equipo de salud debe garantizar la continuidad de la atención^(2)^.

En Brasil, la Estrategia de Salud Familiar (ESF), en el contexto de la Atención Primaria de Salud (APS), desempeña un papel fundamental en la ampliación del acceso y la promoción de una atención prenatal de calidad, y se considera una estrategia esencial para el control y la prevención de la SG y la SC^(4)^.

Aunque el acceso a la atención prenatal está ampliamente garantizado en Brasil, un estudio señala deficiencias en la calidad de la atención ofrecida, lo que genera situaciones preocupantes de vulnerabilidad ^(5)^. Una investigación realizada en Rio Grande do Sul evidenció que, incluso entre las gestantes que acudieron a la atención prenatal, la inadecuación del tratamiento (52%) y la ausencia de este (32,4%) siguen siendo factores de vulnerabilidad a la SC, al igual que la falta de tratamiento de las parejas sexuales, lo cual constituye uno de los principales obstáculos para el control efectivo de la sífilis^(6)^.

La falta de acceso a servicios de salud de calidad, especialmente en lo que se refiere a la realización de pruebas de detección e intervenciones terapéuticas efectivas, sigue siendo un factor significativo de vulnerabilidad para la elevada prevalencia de esta afección en diversas regiones del mundo^(7)^.

Aunque la SG no figura entre las 20 enfermedades tropicales desatendidas, forma parte de las estrategias y metas establecidas por la OMS^(8)^ para la eliminación del descuido y el logro de los Objetivos de Desarrollo Sostenible (ODS) para 2030. La literatura científica internacional, sin embargo, reconoce la SG como una enfermedad desatendida^(9)^, fuertemente asociada a condiciones de vulnerabilidad que afectan a poblaciones específicas^(10,11)^.

Es importante destacar que la definición de vulnerabilidad se relaciona con un conjunto de aspectos individuales, colectivos y contextuales que provocan una mayor susceptibilidad a la infección y a la enfermedad, siendo indisociable, en mayor o menor medida, del acceso a recursos para garantizar su protección^(12)^.

La vulnerabilidad en salud se entiende como una condición humana, derivada de la interacción entre el sujeto y lo social, a partir de las relaciones de poder, y que puede encontrarse en una situación de precariedad cuando el empoderamiento del sujeto o del colectivo no se está experimentando en ese momento ^(13)^. Estas fragilidades abarcan aspectos sociodemográficos, entre los que se encuentran el acceso a los servicios de salud, las condiciones socioeconómicas y la dimensión cultural, que provienen de la propia sociedad e interfieren en el movimiento de individuos y grupos dentro de una jerarquía social^(11)^.

Estudios nacionales e internacionales evidencian que los determinantes sociales desfavorables, como el bajo nivel educativo, los bajos ingresos y la migración, así como el déficit en la educación en salud, contribuyen a una mayor vulnerabilidad frente a la SG y sus consecuencias^(14,15)^. En este contexto, el análisis de las vulnerabilidades se vuelve fundamental para comprender los factores que influyen en las **altas tasas de incidencia y prevalencia**, la persistencia de la SG y la transmisión vertical. Estas vulnerabilidades pueden entenderse a partir de tres dimensiones interdependientes: la individual, que involucra aspectos biológicos, conductuales y emocionales; la social, relacionada con el contexto y las relaciones interpersonales; y la programática, que se refiere a las políticas públicas, los programas y los servicios de salud disponibles^(16)^.

Estas dimensiones abarcan aspectos individuales relacionados con la exposición y la susceptibilidad a la afección en cuestión; características contextuales y de los vínculos sociales establecidos; así como la forma en que las tecnologías expresadas en políticas, programas, servicios y acciones operan en estos contextos e influyen en las situaciones^(16)^. De este modo, estas tres dimensiones analíticas contribuyen a comprender la relación entre el individuo y la vulnerabilidad.

Para abordar eficazmente la SG, es crucial identificar los factores relacionados con las vulnerabilidades que favorecen su persistencia e implementar estrategias de salud pública que tengan en cuenta las vulnerabilidades y promuevan el acceso equitativo a los servicios de salud. Para ello, se requiere una estructura de análisis que incorpore el comportamiento individual, el ámbito colectivo y el social^(16)^.

Ante esta problemática, surge la pregunta: ¿cuál es la percepción de las mujeres diagnosticadas con SG sobre el seguimiento prenatal realizado en la ESF y las posibles vulnerabilidades individuales, sociales y programáticas asociadas a dicho seguimiento?

La relevancia de este estudio se justifica por el impacto de la SC en el escenario brasileño, considerando las consecuencias inmediatas y a largo plazo para los recién y la salud neonatal. En este contexto, el presente estudio tiene como objetivo analizar la percepción de las mujeres diagnosticadas con SG sobre el seguimiento prenatal en la ESF y discutir las vulnerabilidades individuales, sociales y programáticas.

## MÉTODO

### Tipo de Estudio

Se trata de una investigación descriptiva, de enfoque cualitativo, orientada por las directrices de los *Consolidated Criteria for Reporting Qualitative Research* (COREQ)^(17)^.

### Lugar, Población y Criterios de Selección

El campo de investigación se llevó a cabo en el Área de Planificación (AP) 5.3, ubicada en la zona oeste del municipio de Río de Janeiro (Brasil), en los hogares de mujeres notificadas con SG en dos Clínicas de Familia, ubicadas en el barrio de Santa Cruz. Cabe destacar que la elección de esta área para la investigación se justifica por el hecho de ser una zona con alta incidencia de casos de SG, según datos extraídos del Sistema de Información de Enfermedades de Notificación (SINAN). La investigación fue realizada exclusivamente por la investigadora, una enfermera que trabaja en la APS y conoce bien el territorio estudiado, lo que facilitó el acercamiento a las participantes y la comprensión de las experiencias relatadas. El contacto con las participantes se llevó a cabo de manera ética, empática y respetuosa, garantizando la confidencialidad de la información y la neutralidad analítica en la interpretación de los resultados.

Participaron en el estudio 20 mujeres a las que se les había diagnosticado SG, cumpliendo con los criterios de inclusión: edad mínima de 18 años, que recibieron seguimiento prenatal en la ESF de la AP 5.3 y notificaciones de SG entre los años 2016 y 2021. Este período corresponde a la implementación de la Agenda de Acciones Estratégicas para la Reducción de la Sífilis en Brasil, que priorizó la integración de instituciones de enseñanza e investigación para ampliar las estrategias de lucha contra la enfermedad ^(18)^. Se excluyó a las mujeres notificadas en el SINAN que no residían en el territorio del estudio.

### Recolección de Datos

La recolección de datos se llevó a cabo entre agosto de 2023 y marzo de 2024, y la selección de las participantes se realizó a partir de la tabla de casos notificados proporcionada por la División de Vigilancia en Salud de la Coordinación de APS de la AP 5.3. Dicha tabla contenía un total de 67 mujeres y algunos datos como edad, dirección y teléfono. De ese total, 19 no cumplían con los criterios de inclusión, 13 ya no vivían en la dirección informada, cuatro no quisieron participar en la investigación por motivos personales y 11 no respondieron a las llamadas telefónicas. Así, con 20 participantes se programaron las visitas domiciliarias, según la preferencia de día y horario de las mujeres, y la recolección de datos se realizó en los hogares de las participantes, en habitaciones individuales para garantizar la privacidad de la información, con la presencia únicamente de la investigadora.

La técnica de recolección de datos utilizada fue un formulario con preguntas cerradas para trazar la caracterización sociodemográfica y los datos referentes al diagnóstico de la SG. El instrumento contenía los siguientes datos: edad, nivel educativo, raza/color autodeclarado, religión, profesión/ocupación, ingreso familiar, prestaciones sociales. En cuanto al diagnóstico de SG, los datos recopilados fueron: edad gestacional al inicio de la atención prenatal, número de consultas, momento del diagnóstico de sífilis, tratamiento realizado y tratamiento de las parejas sexuales.

La segunda etapa de recolección de datos consistió en una entrevista individual semiestructurada en profundidad que abarcó los siguientes temas: percepción sobre el seguimiento prenatal tras el diagnóstico de sífilis en el embarazo, cómo fue recibir el diagnóstico, cómo fueron las explicaciones/orientaciones en relación con la SG y percepción respecto al acceso a los servicios de salud y seguimiento en relación con el diagnóstico, el tratamiento de la SG y la prevención de la SC. Cabe destacar que se realizaron dos entrevistas piloto con gestantes diagnosticadas con SC fuera del criterio de inclusión del marco temporal, con el fin de ajustar el guion de la entrevista, pero que no se contabilizaron en el total de la muestra.

Las entrevistas se grabaron, tras la lectura y firma del Consentimiento Libre e Informado, previa autorización, mediante una aplicación en un dispositivo móvil, con una duración media de 30 minutos y, posteriormente, se transcribieron íntegramente para su análisis.

### Análisis y Tratamiento de los Datos

El proceso de análisis de datos de las entrevistas se llevó a cabo utilizando el software IRAMUTEQ (*Interface de R pour les Analyses Multidimensionnelles de Textes et de Questionnaires*), mediante análisis de contenido léxico. Este software permite integrar métodos estadísticos y análisis subjetivos, con representaciones gráficas de fácil interpretación y aplicabilidad en bases de datos extensas, siempre que el corpus textual esté debidamente preparado^(19)^. El corpus textual se organizó, abarcando los conceptos de corpus, texto y segmento de texto (ST). El corpus abarcó el conjunto de las 20 entrevistas, cada una tratada como texto, definiéndose el ST como unidad analítica con un tamaño aproximado de tres líneas, ajustado automáticamente por IRAMUTEQ^(19)^.

Las líneas de comando, precedidas por cuatro asteriscos (****) y seguidas de códigos específicos, identificaron a las participantes utilizando la letra M de mujer seguida del número (M_1 a M_20) y las variables estudiadas fueron edad, tener pareja, haber realizado tratamiento y nivel educativo. Todas estas etapas aseguraron el rigor metodológico, el anonimato de las participantes y la organización necesaria para el procesamiento y análisis de los datos.

Este estudio utilizó el Análisis por Clasificación Jerárquica Descendente (CJD), que agrupa los ST basándose en vocabularios similares, fragmentando el corpus textual de acuerdo con la frecuencia de formas reducidas^(19)^. El objetivo principal es identificar clases de vocabulario que presenten homogeneidad interna y heterogeneidad entre sí, lo que permite el análisis detallado de ST mediante pruebas de Chi-cuadrado (chi^2^) para calcular distancias y proximidades entre los términos^(19)^.

El análisis de la CJD dividió el corpus textual en cinco clases temáticas, lo que supuso un aprovechamiento del 81,01% del material analizado. Esta distribución reveló dos bloques temáticos, siendo el bloque 1 denominado “La sífilis gestacional y el seguimiento prenatal: la presencia de vulnerabilidades”, formado por las clases 1, 4 y 5. Este bloque fue el que respondió al objetivo de este estudio, abordando los aspectos relacionados con la identificación de la sífilis durante la gestación, la percepción de las participantes sobre la atención recibida y las vulnerabilidades individuales, programáticas y sociales en el seguimiento prenatal.

Los resultados brutos proporcionados por el software fueron tratados e interpretados, buscando atribuir significados y validez a las clases encontradas. El análisis incluyó deducciones lógicas, descripción y contextualización basadas en la literatura científica relevante, lo que permitió una interpretación más profunda de los discursos y una comprensión de los mensajes implícitos en los vocabularios identificados, que fueron analizados y nombrados con base en los referentes teóricos sobre la SG y las dimensiones de las vulnerabilidades.

### Aspectos Éticos

El estudio se llevó a cabo de conformidad con la Resolución n.º 466/2012 del Consejo Nacional de Salud/MS, que regula las investigaciones con seres humanos, y con la Resolución n.º 580/2018, aplicable a las investigaciones realizadas en instituciones del Sistema Único de Salud (SUS). Fue evaluado y aprobado por el Comité de Ética en Investigación de la Universidad Federal de Río de Janeiro – Escuela de Enfermería Anna Nery/Hospital Escuela São Francisco de Assis, CAAE 66485023.2.0000. 5238 y número de dictamen 6.175.541, y por el Comité de Ética en Investigación de la Secretaría Municipal de Salud de Río de Janeiro, CAAE 66485023.2.3001.5279 y número de dictamen 6.210.457.

## RESULTADOS

Las participantes de este estudio fueron 20 mujeres, con edades comprendidas entre los 20 y los 48 años, siendo el grupo de edad de 30 a 39 años el más prevalente (nueve mujeres). En cuanto a la autodeclaración de raza/color, 13 se identificaron como mestizas y cinco como negras, lo que suma un total de 18 mujeres negras. El nivel educativo fue predominantemente bajo, con ocho mujeres que no habían completado la educación secundaria. En cuanto a la religión, 12 declararon no tener vínculos religiosos, mientras que ocho afirmaron practicar religiones cristianas, siendo seis evangélicas y dos católicas. En lo que respecta a la situación conyugal, nueve eran solteras sin pareja, mientras que 11 convivían con una pareja. Entre las solteras, siete vivían solo con sus hijos, mientras que las mujeres con pareja residían, en su mayoría, con su esposo e hijos. El análisis de la ocupación reveló que 14 estaban desempleadas y seis realizaban actividades remuneradas. El ingreso familiar varió entre menos de un salario mínimo y cuatro salarios mínimos, y 10 sobrevivían con menos de un salario mínimo. Además, 13 recibían algún beneficio social del gobierno.

En cuanto al seguimiento prenatal, 12 lo iniciaron antes de las 12 semanas de gestación, y seis no recordaban la edad gestacional al inicio. El número de consultas varió de cuatro a 14, con nueve mujeres que realizaron menos de seis consultas, cifra inferior a la recomendada por el Ministerio de Salud^(2).^ Además, el diagnóstico de sífilis se produjo mayoritariamente durante la gestación en 15 mujeres, mientras que cinco fueron diagnosticadas solo en el momento del parto o después del nacimiento del hijo. Entre las gestantes diagnosticadas, 15 recibieron tratamiento con bencilpenicilina, dos utilizaron otros medicamentos debido a alergias y una no recibió tratamiento. En lo que respecta a las parejas sexuales, 12 afirmaron que la pareja se sometió al tratamiento, mientras que 8 participantes declararon que las parejas no aceptaron utilizar la medicación.

En los resultados de las entrevistas, en relación con el análisis de la CJD del software IRAMUTEQ para este artículo, se analizaron las clases correspondientes al bloque 1, clases 1, 4 y 5, y se organizaron según las vulnerabilidades presentadas.

De este modo, las clases generadas por IRAMUTEQ se interpretaron a la luz de las tres dimensiones de la vulnerabilidad en salud: individual, social y programática. Así, una misma clase puede contener contenidos que dialogan con más de una dimensión, presentándose según el enfoque analítico predominante en cada eje.

### Vulnerabilidad Individual

#### Clase 1 – Diagnóstico de Sífilis Gestacional y la (Des) Información: lo Inesperado de la Revelación

La Clase 1 estuvo compuesta por 9 ST, lo que representa el 14,06% del material clasificado para el análisis. Los léxicos diagnóstico (chi^2^ – 16,19), inicio (chi^2^ – 13,11) y asustada (chi^2^ – 7,21) asociados a los ST evidenciaron los sentimientos de aprensión y susto experimentados por las participantes al recibir el diagnóstico de sífilis durante la gestación. Los ST que se destacan a continuación revelan las emociones negativas experimentadas por las participantes, ilustrando las reacciones iniciales ante el diagnóstico de SG:

*Recibir el diagnóstico de sífilis fue horrible, fue molesto, malo* [...]*, yo también me asusté mucho, nunca había tenido sífilis y no sabía lo que era (M4).*

[...] *recibir el diagnóstico de sífilis fue difícil* [...] *porque al principio no lo aceptaba,* [...] *para mí fue un poco perturbador, aterrador (M5).*

Otros ST analizados revelaron que las participantes no tenían conocimiento previo sobre su condición de infectadas, ni comprendían qué era la sífilis, lo que demuestra la presencia de vulnerabilidad individual.

*Cuando me diagnosticaron no sabía qué era,* [...] *me enteré después de que hablaran conmigo (M3).*

*Cuando me diagnosticaron sífilis no lo entendía,* [...] *fui a investigar y fue entonces cuando lo entendí (M18).*

### Vulnerabilidad Social

#### Clase 4 – Determinantes Sociales Desfavorables que Afectan la Adherencia al Seguimiento Prenatal

La Clase 4 estuvo compuesta por 13 ST, lo que representa el 20,31% del material analizado. Los léxicos prenatal (chi^2^ – 17,44) y seguimiento (chi^2^ – 10,23) asociados a los ST de mayor significancia presentan contenidos que señalan las dificultades que las mujeres relataron para dar continuidad al seguimiento prenatal, como vivir lejos, comprender la importancia de la atención prenatal ante el diagnóstico de SG y la falta de una red de apoyo, lo que da lugar a situaciones de vulnerabilidad social.

*En mi seguimiento prenatal no me atendieron mejor porque ya no fui a las consultas,* [...] *vinieron a visitarme y me dijeron que volviera a las consultas,* [...] *pero no fui porque estaba muy lejos, me costaba mucho conseguir transporte (M10).*

*En ese momento era muy joven, no me preocupaba mucho,* [...] *fui a la primera consulta cuando descubrí el embarazo, y después pasé bastante tiempo sin ir. No tenía ayuda de nadie en casa (M 20).*

### Vulnerabilidad Programática

#### Clase 4: Seguimiento de la Sg: Percepción De Un Prenatal “Normal”

También en la Clase 4, a través del léxico “normal” (chi^2^ – 25,97), surgió la percepción del seguimiento prenatal como algo común, incluso ante un cuadro de riesgo como la SG. Esta clasificación, posiblemente, se basa en el desconocimiento sobre la problemática vivida y sus consecuencias, ya que, como destacan las ST, no tuvieron información sobre la SG;

*Mi seguimiento prenatal fue normal,* [...] *fue mi primer embarazo (M2).*

*Mi seguimiento prenatal fue normal,* [...] *una vez al mes y luego ya era cada quince días*[...]*hice el seguimiento y tomé la medicina que me recetaron, pero no me explicaban mucho (M9).*

### Clase 5 – Carencias Percibidas en el Seguimiento Prenatal

La Clase 5 estuvo compuesta por 14 ST, lo que representa el 21,88% del material clasificado para el análisis, y los léxicos con mayor significación estadística asociados a esta clase fueron: examen (chi^2^ – 48,13), clínica de familia (chi^2^ – 18,74) y sangre (chi^2^ – 14,63). Los ST específicos de esta clase muestran que las mujeres señalaron que el seguimiento prenatal se percibió como una atención mínima, solo medicamentosa, lo que demuestra la presencia de vulnerabilidad programática:

*Solo me dieron la medicación,* [...] *luego solo me pidieron un análisis de sangre,* [...] *no pidieron nada más y no dijeron nada más (M5).*

*En los servicios de salud solo me dijeron que tendría que ponerme la vacuna, pero no me explicaron cómo era, qué iba a pasar* [...] *y solo me hicieron un análisis de sangre (M18).*

Reforzando la presencia de la vulnerabilidad programática, otras ST de la Clase 5 también presentaron percepciones sobre la atención recibida, específicamente en las Clínicas de Familia donde realizaron las consultas prenatales, evidenciando las debilidades en cuanto a la estructura, la acogida, la organización, el vínculo y la calidad de los servicios de salud ofrecidos.

*No me traté la sífilis en la clínica de familia porque mi examen al inicio del embarazo dio negativo y al final del embarazo dio un resultado dudoso. ¡Muy desorganizado! (M12)*

*No me gustó, no recibí ninguna orientación sobre la sífilis durante el embarazo, cómo prevenirla y cómo se transmite al feto (M18).*

### Clase 5 – Detección de la Sífilis Mediante el SC: Desenlace Desfavorable

Ante las deficiencias percibidas en el seguimiento prenatal, algunos ST muestran un desenlace desfavorable, como la detección de la sífilis solo en la maternidad, al nacer el hijo, es decir, la constatación del SC. Algunas mujeres percibieron fallas en relación con la atención prenatal, tal como señalan los relatos de experiencia de la clase 4:

*La sífilis no se detectó en los exámenes desde las 5 semanas hasta mis 32 semanas.* [...] *Me hicieron seguimiento, pero como no se había detectado en la clínica de familia, solo se detectó en el hospital. Ya con mi hijo en la UTI con sífilis (M16).*

*Me pareció que mi seguimiento prenatal no fue bueno; de lo contrario, no habría perdido a mi hijo por sífilis. Allí en la clínica, la doctora intentó escuchar el corazón del bebé con tres aparatos y no lo logró. Yo pensaba que tenía que enviarme al hospital, a algún lugar, pero ella dijo que era normal.*

## DISCUSIÓN

A partir de la información recopilada sobre la percepción de la SG, se buscó analizar, desde la perspectiva de las vulnerabilidades —que resultan de la interacción de factores individuales, colectivos y contextuales^(1)^—, la susceptibilidad a esta infección y al proceso de enfermarse. De este modo, el concepto de vulnerabilidad trasciende la dimensión biológica e incorpora determinantes sociales, económicos, culturales y programáticos, ofreciendo una base teórica fundamental para políticas y prácticas de salud integradoras y equitativas^(20)^.

Los resultados de este estudio refuerzan que la sífilis afecta a mujeres de diferentes grupos de edad, destacando su predominio entre las mujeres negras, con bajo nivel educativo y menor poder adquisitivo. Este perfil refleja vulnerabilidades sociales marcadas por el racismo estructural y las desigualdades en el acceso a los servicios de salud. Estos hallazgos corroboran evidencias anteriores que indican una mayor exposición de este perfil de mujeres a las infecciones de transmisión sexual (ITS), debido a factores socioeconómicos y culturales^(21,22)^. El bajo nivel educativo y las precarias condiciones económicas de las participantes se revelan como factores determinantes de las barreras para acceder a la atención prenatal y al cumplimiento del tratamiento contra la sífilis, en consonancia con los resultados de otros estudios^(23,24)^. Estos factores se ven agravados por una atención prenatal inadecuada y por la escasez de orientaciones preventivas y terapéuticas, lo que refuerza la necesidad de estrategias integradas que consideren las especificidades culturales y sociales de las mujeres en contextos de mayor vulnerabilidad^(25)^, como es el caso de las participantes de este estudio.

La sífilis, al ser una ITS, presenta desafíos significativos durante la gestación, generando tensiones biológicas, emocionales y sociales. En el ámbito de la salud, la vulnerabilidad se entiende como una condición humana que surge de la interacción dinámica entre el sujeto y el contexto social, impregnada por las relaciones de poder. Esta condición se vuelve crítica cuando hay precariedad, caracterizada por la ausencia de empoderamiento individual o colectivo. La precariedad, en este sentido, se manifiesta en problemas psíquicos, físicos, sociales o relacionales. Por otro lado, el empoderamiento se relaciona con el fortalecimiento de redes de apoyo, prácticas reflexivas, espiritualidad, sentimientos positivos, promoción de la salud y resiliencia^(9)^, prácticas que no fueron relatadas ni experimentadas por las participantes, lo que refuerza las situaciones de vulnerabilidad individual y social.

Ante el diagnóstico de la SG, las participantes expresaron sentimientos inesperados: sorpresa, aprensión y ansiedad, a menudo intensificados por el desconocimiento sobre esta infección y sus implicaciones para la salud materno-fetal. Este resultado pone de manifiesto la vulnerabilidad individual relacionada con los sentimientos negativos experimentados ante algo desconocido y oscuro^(20)^, lo que compromete la adhesión integral al proceso de tratamiento, tal como también se describe en otro estudio^(23)^.

La desinformación sobre la sífilis se identificó como una barrera relevante para la prevención y el manejo de la infección, lo que impacta negativamente en el inicio de la atención prenatal y, en consecuencia, en el diagnóstico precoz. Estudios realizados en Brasil y a nivel internacional destacan la correlación entre el bajo nivel educativo, la falta de conocimiento y de acceso a información confiable sobre la sífilis y la falta de vínculo con los profesionales de la salud como factores primordiales para las altas incidencias y prevalencias de esta ITS^(25,26)^.

En este contexto, las acciones educativas participativas y con mayor integración surgen como estrategias fundamentales para fortalecer el autocuidado y reducir las vulnerabilidades^(16,26)^. Estos hallazgos corroboran un estudio realizado en los Estados Unidos que señala la necesidad de ampliar las pruebas y el tratamiento de las gestantes y sus parejas, mejorar el acceso y la vinculación a una atención materno-infantil eficaz, pero principalmente, fortalecer las acciones de educación en salud^(27)^.

La desigualdad de género es, además, uno de los factores de mayor vulnerabilidad social de las mujeres frente a las ITS, incluida la SG. Las cuestiones de género, que colocan a las mujeres en una posición de inferioridad y sumisión respecto a los hombres, las privan del poder de decisión, como por ejemplo, el uso de preservativos durante las actividades sexuales y el tratamiento de la sífilis^(24)^, tal como se puede verificar en este estudio, ya que solo 12 parejas sexuales se adhirieron al tratamiento. Cuestiones como la dependencia económica, la dificultad para dialogar con las parejas, el miedo a la violencia doméstica y la naturalización de la dominación masculina impiden que las mujeres asuman un papel protagonista en su salud sexual y reproductiva, lo que dificulta la prevención de la SG. En este contexto, un enfoque científico, acogedor, sin juicios y humanizado por parte de los profesionales debe considerar la intersección de los factores biológicos, sociales y culturales para comprender la desigualdad de género en el contexto de la SG, destacando la necesidad de acciones que promuevan el empoderamiento femenino^(28)^.

La percepción de la atención prenatal por parte de las entrevistadas presentó debilidades significativas, principalmente en relación con la gestión, que se entendió como inadecuada, desorganizada y desproporcionada respecto a los riesgos asociados a la SG. Destacan que fue un tema abordado únicamente de manera medicalizada y fragmentada, solo cuando se confirmó el diagnóstico, perpetuando así el desconocimiento sobre esta ITS y dificultando la toma de decisiones preventivas, lo que refuerza las situaciones de vulnerabilidad y, en consecuencia, genera resultados desfavorables.

Estas limitaciones pusieron de manifiesto las vulnerabilidades programáticas en los servicios de salud, caracterizadas por la ausencia de orientaciones eficaces y conductas apropiadas. La brecha en el vínculo entre los profesionales de la salud y las gestantes refleja la necesidad de mejorar la calidad de la atención prenatal, promoviendo orientaciones sobre prevención, diagnóstico y tratamiento, de manera continua y con seguimiento^(29)^. La vulnerabilidad programática se refiere a las inversiones en acciones y programas de información y educación preventivas, así como al acceso a servicios sociales y de salud de alta calidad, diseñados de manera democrática, con retroalimentación y evaluación periódicas, y sin discriminación institucional^(12)^.

La cobertura prenatal en Brasil, aunque elevada, presenta desigualdades regionales y limitaciones significativas en la calidad de la atención, lo que compromete la detección temprana y el manejo clínico de la SG, perpetuando tasas preocupantes de SC^(28,29)^. Los resultados de este estudio evidencian que, en diversas situaciones, el diagnóstico de la SG se produce solo en el momento del parto, lo que revela fallas críticas en el seguimiento prenatal, cuya atención aún se concentra predominantemente en la realización de exámenes de laboratorio y en la prescripción de medicamentos, sin garantizar la integralidad y la efectividad de la atención. Esta constatación refuerza la necesidad de replantear la práctica clínica, priorizando no solo la identificación temprana del problema, sino también el seguimiento continuo de las gestantes y sus parejas.

La eficacia de las intervenciones prenatales depende de esfuerzos estructurales y programáticos que superen las desigualdades sociales, políticas, culturales y económicas, capaces de influir directamente en la vulnerabilidad de las mujeres frente a la SG^(20,24)^. En este contexto, los servicios de APS deben reorientarse para integrar estrategias de atención que consideren no solo el cuadro clínico, sino también las condiciones sociales e individuales de las gestantes, como el bajo nivel socioeconómico, la dificultad de acceso a la información y a los tratamientos, la ausencia de una red de apoyo y otras barreras que aumentan el riesgo de transmisión vertical de la infección^(22,29)^.

Sin embargo, este estudio se llevó a cabo en un territorio históricamente marcado por profundas desigualdades sociales, condición que justifica su elección como campo empírico en consonancia con el objetivo investigado. Al analizar la percepción de las mujeres diagnosticadas con SG sobre el seguimiento prenatal, resulta imprescindible comprender este fenómeno en contextos en los que las vulnerabilidades individuales, sociales y programáticas se manifiestan de forma más intensa, lo que incide directamente en el acceso a los servicios de salud, la calidad de la atención ofrecida y los resultados materno-infantiles.

La atención prenatal debe ir más allá de la mera solicitud y evaluación de pruebas de detección y diagnóstico de la sífilis o del seguimiento del tratamiento. Es esencial que los profesionales de la salud asuman un papel educativo con la gestante y su familia, basado en la acogida y el acompañamiento continuo, ofreciendo apoyo y orientación sobre los riesgos de la infección para la salud materna y fetal. Además, el fortalecimiento de los vínculos entre el equipo de salud, la gestante y sus familiares genera repercusiones positivas, como una mayor adherencia al plan terapéutico propuesto. Esta relación de confianza favorece la regularidad en las consultas y permite un seguimiento más efectivo, con un monitoreo sistemático y organizado, lo que tiene un impacto positivo en la evolución de la gestación^(29)^. Estos son aspectos que no se percibieron en los comentarios de las participantes de este estudio, lo que refuerza la presencia de la vulnerabilidad programática.

Ante la presencia de situaciones de vulnerabilidad, la educación continua para los profesionales de la salud es esencial para mejorar el manejo de toda la problemática de la SG más allá del diagnóstico y el tratamiento. Las tecnologías ligeras y las prácticas educativas en salud, como cursos y capacitaciones para calificar al equipo de salud, la elaboración de herramientas visuales que detallen el flujo de diagnóstico, tratamiento y seguimiento en todos los niveles y la atención de salud, talleres educativos territoriales con recursos pedagógicos culturalmente sensibles, actualizados y adaptados a las diversas realidades, el uso de las Líneas de Cuidado y los sistemas de información integrados; la coordinación entre servicios y el fortalecimiento de equipos multiprofesionales, así como estrategias que involucren a la comunidad y a las parejas afectivo-sexuales, son acciones fundamentales para minimizar las vulnerabilidades de las mujeres diagnosticadas con SG y promover la calidad de la atención a esta población^(29)^.

Como limitación del estudio, cabe destacar la dependencia de relatos retrospectivos sujetos a sesgos de memoria y el enfoque en una única AP, lo que imposibilita la generalización de los resultados. Sin embargo, la investigación ofrece una comprensión profunda y singular de las experiencias de las mujeres entrevistadas, lo que permite reflexiones esenciales para la mejora de las políticas y prácticas de salud frente a la lucha contra la problemática de la SG y la SC.

## CONCLUSIÓN

El estudio pone de manifiesto la complejidad de la SG y las deficiencias en el manejo de esta afección en los servicios de APS. Destacan las vulnerabilidades individuales, sociales y programáticas a las que se enfrentan las mujeres afectadas por la SG, las cuales demostraron comprometer tanto la detección temprana como el tratamiento oportuno durante el periodo prenatal, lo que da lugar a la transmisión vertical de la enfermedad.

Esta situación resalta la urgencia de una atención prenatal humanizada, con una acogida respetuosa y un seguimiento continuo y riguroso, que fortalezca el vínculo entre las gestantes y los profesionales de la salud, siendo fundamental que esta atención comprenda el contexto sociocultural y económico de las mujeres, promoviendo acciones integradas que superen las vulnerabilidades señaladas.

El fortalecimiento de los servicios de APS con tecnologías ligeras y acciones educativas asociadas a la humanización de la atención se vuelve urgente y necesario con el fin de reducir los índices de SG y, en consecuencia, de SC, promoviendo así la calidad de la salud sexual y reproductiva.

## Data Availability

Todo el conjunto de datos que apoya los resultados de este estudio fue publicado en el propio artículo.
